# MicroRNAs in Microglia: How do MicroRNAs Affect Activation, Inflammation, Polarization of Microglia and Mediate the Interaction Between Microglia and Glioma?

**DOI:** 10.3389/fnmol.2019.00125

**Published:** 2019-05-10

**Authors:** Yawei Guo, Wenming Hong, Xinming Wang, Pengying Zhang, Heinrich Körner, Jiajie Tu, Wei Wei

**Affiliations:** ^1^Key Laboratory of Anti-inflammatory and Immune Medicine, Anhui Collaborative Innovation Center of Anti-inflammatory and Immune Medicine, Institute of Clinical Pharmacology, Anhui Medical University, Ministry of Education, Hefei, China; ^2^Department of Neurosurgery, The First Affiliated Hospital of Anhui Medical University, Hefei, China

**Keywords:** microRNA, microglia, activation, inflammation, polarization, glioma

## Abstract

The essential roles of microglia in maintaining homeostasis in the healthy brain and contributing to neuropathology are well documented. Emerging evidence suggests that epigenetic modulation regulates microglial behavior in both physiological and pathological conditions. MicroRNAs (miRNAs) are short, non-coding epigenetic regulators that repress target gene expression mostly *via* binding to 3′-untranslated region (3′-UTR) of mRNA in a Dicer-dependent manner. Dysregulation of certain miRNAs can contribute to microglial hyper-activation, persistent neuroinflammation, and abnormal macrophage polarization in the brain. These abnormal conditions can support the pathogenesis of neurological disorders such as glioma, Alzheimer’s disease (AD), amyotrophic lateral sclerosis (ALS), stroke, ischemia, and spinal cord injury (SCI). However, the roles of miRNAs in microglia in health and neurological disease have not been systematically summarized. This review will first report the role of Dicer, a key endoribonulease that is responsible for most miRNA biogenesis in microglia. Second, we will focus on recent research about the function of miRNAs in activation, inflammation and polarization of microglia, respectively. In addition, potential crosstalk between microglia and glioma cells *via* miRNAs will be discussed in this part. Finally, the role of two essential miRNAs, miR-124, and miR-155, in microglia will be highlighted.

## Introduction

Microglia are resident macrophages in the brain that contribute to immunological homeostasis in the central nervous system (CNS; Paolicelli et al., 2011; Schafer et al., 2012) and become activated during CNS pathology (Wake et al., 2009). To maintain homeostasis in the healthy CNS, microglia participate in brain (Paolicelli et al., 2011) and synaptic development (Tremblay et al., 2011) through active communication with neurons (Schafer et al., 2013). Emerging evidence suggests that microRNAs (miRNAs) have various essential roles in the normal brain (Boudreau et al., 2014) and have been implicated in neuropathological conditions (Brettschneider et al., 2015). MiRNAs repress gene expression by binding to the 3′-untranslated region (3′-UTR; Tu et al., 2015), coding sequence (Fang and Rajewsky, 2011) or 5′UTR (Lee et al., 2009) of target genes. An individual miRNA can target hundreds of genes simultaneously (Hong et al., 2018) which makes its contribution to regulatory mechanism difficult to appreciate.

The specific roles of individual miRNAs in microglia have been extensively investigated (Amici et al., 2017). Activated microglia respond strongly to neurotransmitters (Town et al., 2005). In most pathological conditions of the CNS, microglia are highly activated (Ponomarev et al., 2005), suggesting that the transition from a resting to an activated state may play an important role in neuropathogenesis. Moreover, communication between microglia and neurons in chronic neuroinflammation has also been demonstrated in several neurodegenerative diseases (Suzumura, 2013). Additionally, the role of microglial polarization in M1 (pro-inflammation) and M2 (anti-inflammation) macrophages/microglia has been demonstrated in several CNS diseases (Jha et al., 2016; Geloso et al., 2017; Labandeira-Garcia et al., 2017; Lan et al., 2017). Finally, recent evidence has demonstrated that microglia abundantly infiltrate the microenvironment of glioma, a malignant brain tumor acting as tumor-associated macrophages (Hambardzumyan et al., 2016).

An improved understanding of the interactions between microglia and glioma could promote novel therapeutic strategies for glioma therapy. In this review, we will discuss in detail the dysregulation of miRNAs in microglia in both the healthy brain and CNS pathologies. The roles of specific miRNAs in mediating microglial activation, inflammation, and polarization signaling cascades will be summarized. The potential role of miRNAs in mediating a crosstalk between microglia and glioma and specifically, the roles of two well-studied miRNAs in microglia, miR-124 and miR-155 will be highlighted.

### Dicer KO Microglia

**Figure 1:**
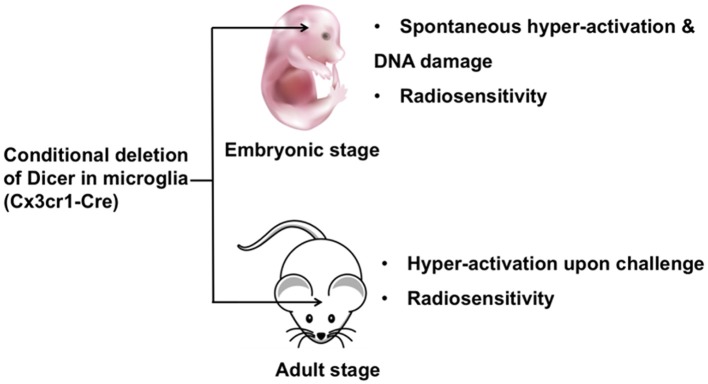
The role of Dicer in embryonic and adult microglia. Ablation of Dicer in microglia during embryonic development leads to spontaneous microglial activation and impaired genome integrity. A conditional Dicer knockout in microglia shows hyper-activation upon stimulation in adult mice. Additionally, Dicer-deletion microglia show increased sensitivity to irradiation in both embryonic and adult stages.

Dicer is responsible for most miRNA biogenesis by acting as endoribonuclease or helicase with an RNase motif and cleaving miRNA precursors into mature miRNAs (Cheloufi et al., 2010). Therefore, most miRNAs are downregulated in Dicer knockout cells. The consequences of this genetic ablation of Dicer are dramatic. A conditional Dicer knockout in microglia (Cx3cr1-Cre) showed hyper-activation upon stimulation. During embryonic development deletion of Dicer led to spontaneous microglial activation and impaired genome integrity. Furthermore, Dicer-negative microglia displayed a shift to an inflammatory state upon peripheral endotoxin challenge, thereby compromising hippocampal neuronal function. Additionally, Dicer-negative microglia showed increased sensitivity to irradiation (Varol et al., 2017). In summary, Dicer knockout microglia exhibited significant changes in normal functions in both prenatal and adult stages (Figure 1), suggesting that individual miRNAs play a role in microglia. It is critical to identify the specific miRNAs that are responsible for microglial dysfunction. Apart from Dicer, biogenesis of some miRNAs is dependent on the protein Argonaute (Cheloufi et al., 2010). Therefore, an investigation of the role of Argonaute in microglia will further clarify the function of miRNAs in brain. In the next section, we will summarize the versatile role of individual miRNAs in microglia.

### The Role of miRNAs in Microglial Activation

Microglial function is essential for neurodegenerative disorders including Parkinson’s disease (PD), multiple sclerosis (MS) and Alzheimer’s disease (AD; Grimmig et al., 2016; Hesse et al., 2016), all of which are closely associated with microglial activation (Lull and Block, 2010; Kandinov et al., 2011; Tanaka et al., 2013; Reemst et al., 2016; Du et al., 2017; Sarlus and Heneka, 2017). In their resting state, microglia show beneficial effects *via* interactions with neurons, thus helping to maintain brain homeostasis. Upon brain injury or infection, microglia transform to an activated state and secrete neurotoxic mediators, such as reactive oxygen species (ROS), tumor necrosis factor alpha (TNF-α), and interleukin-1β (IL-1β; Belarbi et al., 2011; Krishnaswamy and Cooper, 2012; Mishra et al., 2012; Yang et al., 2013) which have harmful effects including disruption of neuronal function/synaptic transmission and neuronal oxidative stress/degeneration resulting in neuronal damage. Therefore, emerging evidence suggests that miRNAs can ameliorate degeneration by inhibiting microglial activation in the brain. A suppression of microglial activation could serve as a potential therapeutic approach to protect neurons and thus, treat or prevent neurodegenerative diseases (Lull and Block, 2010). The following section discusses the effects of miRNAs on microglial activation in preventing neuronal damage.

Intracerebroventricular injection of let-7c-5p mimics reduced infarction volume and ameliorated neurological defects *via* inhibition of microglia activation in a model of cerebral ischemia injury. These effects were mediated by inhibition of caspase-3 (Ni et al., 2015). Furthermore, it has been shown that microglial activation can induce ischemia, a condition that could be repressed by miR-203, which directly targeted MyD88 in microglia. A miR-203 overexpression or a MyD88 knockdown led to repression of NF-κβ signaling and prevented subsequent microglial activation (Yang et al., 2015) ameliorating neuronal injury. Another microRNA, miR-145-5p, was shown to directly bind to the 3′-UTR of the mRNA of Nurr1, a member of the orphan nuclear receptor family that induces neuronal death and consequently, inhibited Nurr1-mediated microglial activation alleviating neuronal injury in acute cerebral ischemic/reperfusion in rats (Xie et al., 2017).

The miR-199b was shown to inhibit the IKKβ-NF-κB signaling pathway and to repress pro-inflammatory cytokines *via* modulation of microglial activation in a rat model of spinal cord injury (SCI; Zhou H. J. et al., 2016). These results imply that miR-199b is a potential therapeutic target for SCI. Additionally, miR-424 was found to be repressed in the plasma of patients with acute ischemic stroke, and in mouse plasma and brain tissues after ischemia. Treatment with miR-424 alleviated brain edema and cerebral infarction size after middle cerebral artery occlusion *via* repression of microglial activation and neuronal apoptosis. It was also demonstrated that miR-424 inhibited ionized calcium-binding adaptor molecule (iba) 1 and reduced pro-inflammatory TNF-α secretion. In addition, *in vitro* experiments further validated that miR-424 inhibited the activity of BV2, a microglial cell line (Zhao et al., 2013). Likewise, miR-7 inhibited microglial NLRP3 inflammasome activation *in vitro*. In contrast, anti-miR-7 induced inflammasome activation. Moreover, stereotactic injection of miR-7 mimics into the mouse striatum ameliorated microglial activation, concomitant with attenuation of dopaminergic neuron degeneration in a mouse model of PD (Zhou Y. et al., 2016) making miR-7 a potential target in PD therapy.

The miR-27a was found to be repressed in lipopolysaccharide (LPS)-activated microglia. It inhibited microglia-produced inflammatory cytokines, including IL-6, IL-1β and TNF-α, and interfered with the expression of TLR4 and interleukin-1 receptor-associated kinase 4 (IRAK4) by directly binding their 3′-UTRs. Downregulation of TLR4 or IRAK4 of the TLR4/MyD88 signaling pathway in microglia inhibited the downstream secretion of inflammatory mediators (Lv et al., 2017). Therefore, miR-27a could regulate LPS-activated production of inflammatory cytokines in microglia by modulating TLR4/IRAK4 activity. Hypoxic conditions were also shown to induce TLR4, to activate microglia, and to reduce miR-181c in the brain. Like miRNA27-a, miR-181c repressed microglial activation by directly targeting TLR4. Moreover, it repressed the production of inflammatory mediators of the NF-κB pathway (Zhang et al., 2015). Thus, miR-181c may play an important role in hypoxic microglial activation and neuroinflammation. The miR-181c-TLR4-NF-κB pathway may be a potential target for cerebral hypoxic diseases. Japanese encephalitis virus (JEV) infection was shown to activate the microglial cell line BV-2 and to upregulate the expression of inducible nitric oxide synthase (iNOS) and cyclooxygenase (COX) 2. miR-29b further increased JEV-induced microglial activation by targeting TNF-α-induced protein (TNFAIP) 3 and increasing the nuclear translocation of NF-κB (Thounaojam et al., 2014).

In amyotrophic lateral sclerosis (ALS), the NF-κB pathway is positively associated with microglial activation and motor neuron injury. The ubiquitin-editing enzyme A20 plays an important role in inhibiting the NF-κB pathway. miR-125b was shown to directly repress A20 and to consequently amplify NF-κB function in microglia in a P2X7 receptor-dependent manner (Parisi et al., 2016). These results highlight an important role for miR-125b in ALS *via* modulation of microglial activation and motor neuron injury. Methamphetamine-induced neurotoxicity is closely linked to microglial activation. Anti-miRNA143-mediated BBC3 induction restored methamphetamine-repressed microglial survival through the modulation of autophagy and apoptosis. BBC3 was also shown to be a direct target of miR-143 in microglia. As such, microinjection of anti-miR-143 into the hippocampus ameliorated methamphetamine-induced microglial activation. A similar result was demonstrated in heterozygous miR-143 mice (Zhang et al., 2016). Future work exploring the specific effects of miR-143-BBC3 on microglial activation is necessary to provide a better understanding of the mechanism of drug addiction. MiR-146a restored learning and memory impairment in an experimental mouse model of Postoperative cognitive dysfunction (POCD) by targeting interleukin-1 receptor-associated kinase 1 (IRAK1) and TNF-receptor-associated factor 6 (TRAF6). A reduced effect of miR-146a on the abnormal activation of microglia in the hippocampus indicates that miR-146a is a potential therapeutic target of POCD (Chen et al., 2019).

**Figure 2:**
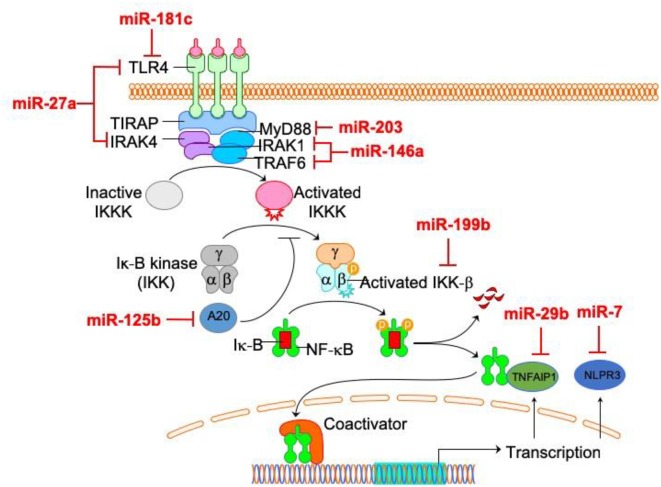
The relationship between microRNAs (miRNAs) and NF-κB pathway in microglia. Among microglia activation-related miRNAs, the direct targets of most miRNAs (miR-27a, miR-181c, miR-203, miR-125b, miR-199, miR-29b, miR-7 and miR-146a) are within NF-κB pathway.

Interestingly, most of these microRNAs are related to the NF-κB pathway in microglia (Figure 2), indicating the central role of NF-κB pathway in microglia activation. Therefore, these findings suggest that miRNAs represent a novel group of NF-κB pathway-related targets regulating microglial activation and brain injury, thus offering a new therapeutic strategy for treating associated neuronal diseases. The modulation of microglial activation by adjusting the interaction between these miRNAs and the NF-κB pathway open new possibilities in the area of therapy for neurodegenerative disorders. By using the tools of gene therapy, miRNA-based “fine-tuning” therapy is a potential method to restore the abnormal activation of microglia.

### The Role of miRNAs in Microglia-Mediated Inflammation

Persistent CNS inflammation could be an underlying cause of cell death in many neurodegenerative diseases, such as HIV-induced dementia, PD, AD, and MS. Thus, microglia-mediated inflammation plays an essential role in neuropathogenesis. Nevertheless, the specific role of microglia in neuron inflammation is a still not entirely characterized. CNS inflammation involving microglia is a normal response to infection. However, when allowed to continue uncontrolled, inflammation may lead to pathological states. Therefore, during the inflammatory process, microglia switch from having a beneficial role to having a detrimental role by secreting cytotoxic molecules, including proinflammatory cytokines and reactive oxygen mediators. Thus, modulating microglia in neuron inflammation is a potential therapeutic strategy for neurodegenerative disease. Indeed, there already exist a number of anti-inflammatory drugs that exert neuronal protective effects by modulating the status of microglia (Ajmone-Cat et al., 2010; Kwon et al., 2013). In recent years, several research groups have investigated the effect of miRNAs on microglia-mediated inflammation. Microglia activation also leads to inflammatory activities, but not all microglia-mediated inflammation is induced by microglia activation. Because we already discuss miRNA in microglia activation in previous section, we only discuss miRNAs in microglia inflammation while not activation.

Both TLR2 and TLR4 activation were shown to increase miR-146a in microglia. In response to TLR2 stimulation, miR-146a disrupted normal inflammatory responses, including NF-kB and JAK-STAT signaling pathways. Two phagocytic mediators of the oxidative burst, CYBA and NOS3, were predicted as direct targets of miR-146a (Saba et al., 2012). In addition, it was reported that in a transgenic reporter mouse, murine microglia specifically lost miR-9. Furthermore, injection of miR-9 vectors into rat brains induced transgene expression in microglia, which permitted direct visualization and isolation of inflammatory microglia without affecting circulation-derived monocytes/macrophages in rats with excitotoxic lesions. As such, miR-9 vectors have been useful in mechanism studies of microglial inflammation (Åkerblom et al., 2013). HIV-1 Tat C exposure repressed miR-17 and induced NOX2/NOX4 and ROS production in human microglial cells. NOX2/NOX4 were shown to be direct targets of miR-17 in microglia (Jadhav et al., 2014). Thus, miR-17 may regulate intracellular ROS generation and subsequent neuroinflammatory responses.

miR-206 was shown to enhance LPS-induced inflammation and cause the release of amyloid-β (an essential protein in AD) in microglia by directly binding to the 3′-UTR of Insulin-like growth factor (IGF) 1. A rescue assay demonstrated that IGF1 exposure could attenuate miR-206-induced inflammation in microglia, indicating that the miR-206/IGF1 signaling pathway may be associated with AD associated microglial inflammation (Xing et al., 2016). miR-26a was rapidly reduced after TLR4 stimulation in microglia and repressed the production of inflammatory cytokines. Furthermore, the ATF2 transcription factor linked to promote inflammation may be a direct target of miR-26a (Kumar et al., 2015), suggesting a role for miR-26a/ATF2-mediated regulation of proinflammatory cytokine production in microglia. miR-93 was significantly inhibited in cerebral ischemia reperfusion (CIR) mice brains. miR-93 overexpression reduced cerebral infarction volume and alleviated neurological deficits in CIR mice. Furthermore, miR-93 inhibited inflammatory responses and decreased the rate of cell apoptosis in CIR mice. Likewise, miR-93 was shown to inhibit IRAK4 and other pro-inflammatory genes in microglia (Tian et al., 2017).

Let-7a is involved in maintaining microglial function in inflammation-mediated injury. During inflammation, Let-7a was shown to inhibit the production of proinflammatory mediators, including iNOS, IL-6, and nitrite, while at the same time inducing anti-inflammatory genes such as brain derived neurotrophic factor (BDNF) and IL-4 in microglia (Cho et al., 2015). Another study showed that the anti-inflammatory factor Apoptosis signal-regulating kinase 1 (ASK1) induced Let-7a activity and consequently activated anti-inflammatory cytokines IL-10 and Mycs in microglia (Song and Lee, 2015). Therefore, Let-7a plays an important role in microglia-related inflammation. miR-32-5p was strongly upregulated in microglia from rats with spinal nerve ligation (SNL). Knockdown of miR-32-5p significantly reduced mechanical allodynia and heat hyperalgesia and decreased the production of inflammatory cytokines in SNL rats. Similarly, miR-32-5p inhibition ameliorated inflammatory cytokine production in LPS-treated microglia. Further investigations demonstrated that Dual-specificity phosphatase 5 (Dusp5) was directly repressed by miR-32. Dusp5 is involved in neuropathic pain and neuroinflammation (Yan et al., 2018). As such, miR-32-5p may induce neuroinflammation and neuropathic pain by modulating Dusp5 activity, suggesting novel therapeutic approaches targeting miR-32-5p-Dusp5 for the treatment of neuropathic pain.

Intracerebral hemorrhage (ICH) decreased miR-367 and increased IRAK4 in primary microglia. IRAK4 is a direct target of miR-367, which may inhibit the NF-κB pathway and block the secretion of related proinflammatory cytokines. Moreover, miR-367 inhibited the production of proinflammatory cytokines and reduced brain edema in ICH mice (Yuan et al., 2015). Therefore, miR-367/IRAK4 represents a potential therapeutic target of neuroinflammation in ICH. Inhibition of miR-204 or SIRT overexpression inhibited LPS-mediated inflammation, proliferation of mouse microglial cells, and promoted apoptosis (Li et al., 2015). Therefore, miR-204 may inhibit microglia-related neuroinflammation in mice by modulating SIRT1. Hyperglycemia- (HG-) Amadori-glycated albumin- (AGA-) treated microglia leads to retinal inflammation, which can cause diabetic retinopathy (DR). In diabetic patients, miR-146b-3p was negatively correlated with adenosine deaminase (ADA) 2, which is a known inducer of retinal inflammation. Overexpression of miR-146b-3p repressed ADA2 and inhibited the production of inflammatory cytokines in microglia (Fulzele et al., 2015).

In summary, the above-mentioned studies stress the role of miRNAs as modulators of neuroinflammation and illustrate their potential as biomarkers and novel therapeutic targets in CNS diseases (Table 1) Thus, miRNAs may provide potential therapeutic strategies for treating cerebral inflammation.

**Table 1:** The effect of microRNAs (miRNAs) on microglia activation and inflammation.

Funtion		miRNA	Target	Reference
Microglia activation	Promotion	miR-145	Nurr1	Xie et al. (2017)
		miR-29b	TNFAIP1	Thounaojam et al. (2014)
		miR-125b	A20	Parisi et al. (2016)
		miR-143	BBC3	Zhang et al. (2016)
	Repression	Let7c-5p	Casepase-3	Ni et al. (2015)
		miR-203	MyD88	Yang et al. (2015)
		miR-199b	IKK	Zhou H. J. et al. (2016)
		miR-424	CCND1/CDC25A/CDK6	Zhao et al. (2013)
		miR-7	Nlrp3	Zhou Y. et al. (2016)
		miR-27a	TLR4/TRAK4	Lv et al. (2017)
		miR-181c	TLR4	Zhang et al. (2015)
		miR-146a	IRAK1/TRAF6	Chen et al. (2019)
Microglia inflammation	Promotion	miR-206	IGF1	Xing et al. (2016)
		miR-36	Dusp5	Yan et al. (2018)
	Repression	miR-146	CYBA/NOS3	Saba et al. (2012)
		miR-17	NOX2/NOX4	Jadhav et al. (2014)
		miR-26a	ATF2	Kumar et al. (2015)
		miR-93	IRAK4	Tian et al. (2017)
		Let-7a	SIRT1	Cho et al. (2015) and Song and Lee (2015)
		miR-367	IRAK4	Yuan et al. (2015)
		miR-204	SIRT1	Li et al. (2015)
		miR-146b	ADA2	Fulzele et al. (2015)

### The Role of miRNAs in Microglial Polarization

Classically, M1 and M2 microglia/macrophages show opposite phenotypes: M1 microglia/macrophages release destructive pro-inflammatory mediators while M2 microglia/macrophages produce protective factors (David and Kroner, 2011). However, the M1 and M2 concept are clearer for *in vitro* studies, while the polarization of microglia/macrophages is more intricate *in vivo* (Moore et al., 2013). M1/M2 classification are therefore a useful concept to identify the specific role of microglia/macrophages in neuropathogenesis (Boche et al., 2013), including SCI, stroke, and TBI (Kigerl et al., 2009; Hu et al., 2012; Wang et al., 2013).

**Figure 3:**
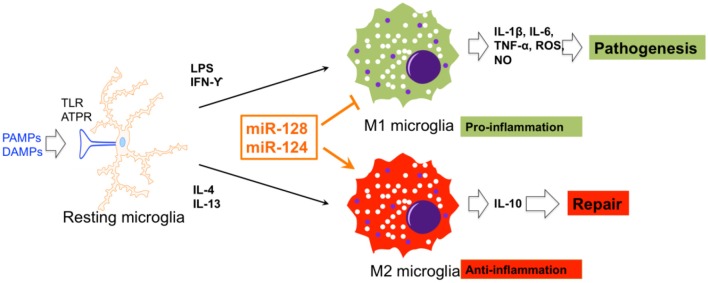
The effects of miRNA on microglial polarization. miR-128 and miR-124 could promote and repress M1 and M2 polarization of microglia, respectively.

In SCI mice, miR-128 was found to be downregulated in microglial BV2 cells. miR-128 markedly promoted the viability of microglia through downregulation of the microglial M1 phenotypic markers, CD86 and CD32, and up-regulation of the M2 phenotypic markers, Arginase (Arg) 1 and CD206. In addition, miR-128 repressed the secretion of inflammatory cytokines. It was also demonstrated that the anti-inflammatory function of miR-128 is mediated by the P38 pathway (Yang et al., 2017). miR-124 promoted neuronal survival and M2-like polarization of microglia. The neuroprotective function of miR-124 was exerted during the first week. Arg1^+^ microglia were positively associated with functional improvement during the same course as miR-124 treatment (Hamzei Taj et al., 2016b). Therefore, miR-124 and miR-128 are two potential targets for functional recovery *via* regulation of microglial polarization (Figure 3).

Overall, the approach to regulate the polarization of microglia by targeting pro-inflammatory or anti-inflammatory miRNAs plays an essential role in plans for a therapy of CNS diseases. However, the main obstacle is how to directly deliver miRNAs to the CNS through the blood brain barrier (BBB) and how to prevent the degradation of exogenous miRNAs by lysosomal activities. With the development of new investigative methods and a better understanding of mechanisms of microglia polarization, microglia polarization based molecular therapy will be potential future research field of neural diseases treatment.

### miRNA-Mediated Interaction Between Microglia and Glioma

**Figure 4:**
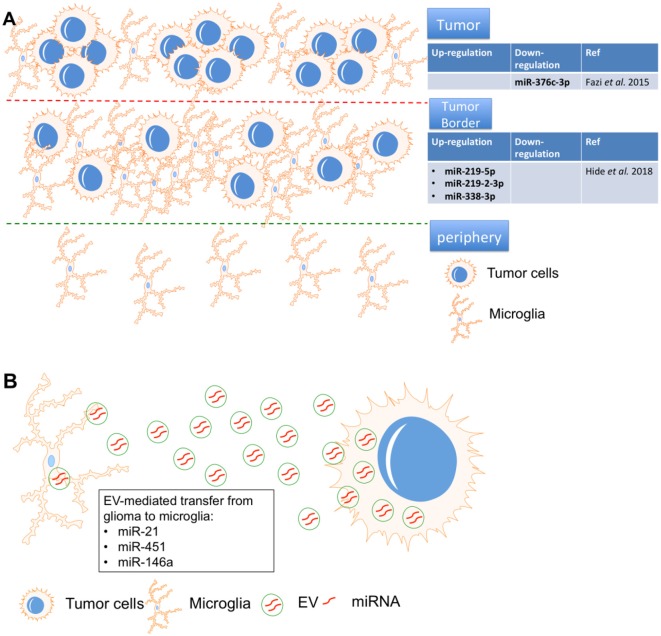
MicroRNA-mediated interactions between microglia and glioma. **(A)** miR-219-5p, miR-219-2-3p, and miR-338-3p were identified as the top three miRNAs in the tumor border and miR-376c-3p was reduced in tumor samples, all were closely associated with macrophages/microglia. **(B)** miR-451, miR-21 and miR-146a in glioma-EVs were found to be transferred to microglia.

In malignant glioma tissues, glioma cells foster a tumor-favorable microenvironment through interactions with other brain cells, including macrophages and microglia. Emerging evidence suggests that intracellular crosstalk between different cellular components within or near the glioma microenvironment occurs *via* transfer of oncogenic factors, including miRNA (Figure 4).

After resection, glioma often recurs due to the chemo-radioresistance of glioma cells. Therefore, identification of the tumor border composition is essential for prognosis in glioma. “miRNAome” expression was compared in tissues from the tumor, tumor border, and periphery by miRNA sequencing. miR-219-5p, miR-219-2-3p, and miR-338-3p were identified as the top three miRNAs in the tumor border, and all were closely associated with macrophages/microglia. Interestingly, the tumor suppressive role of miR-219 has been proven in glioma cells by targeting Sal-like protein (Sall4; Jiang et al., 2017) and epidermal growth factor receptor (EGFR; Rao et al., 2013). Similarly, miR-338 also showed inhibitory effects on glioma cells by repressing C-terminal binding protein 2 (CTBP2; Liu et al., 2017) and Forkhead box d1 (FOXD1; Ma et al., 2018). However, these studies only proved the roles of miR-219 and miR-338 in glioma cells. The crosstalk between glioma cells and microglia *via* these two miRNAs should be systematically investigated in the future.

Macrophages and microglia strongly infiltrate the microenvironment of glioma tumor border, which is a potential target for recurrence (Hide et al., 2018). Using deep sequencing, another group found that the “miRNAome” in peritumoral areas is quite different from that of white matter but shares similarities with tumor tissue. The data revealed that miR-376c-3p was reduced in tumor samples compared to peritumoral tissues and in short-term tumors compared to long-term tumors (Fazi et al., 2015). miR-376c could repress the malignant behavior of glioma cells by repressing FOXP2 (Li et al., 2019) and circulating miR-376c was significantly down-regulated in serum from glioma patients when compared with healthy control (Huang et al., 2017), suggesting that miR-376c is a potential diagnostic marker and treatment target for glioma. Therefore, the specific role of miR-376c the microenvironment of glioma also should be studied.

Due to complicated packaging and uptake by recipient cells, extracellular vesicle (EV)-mediated miRNA transfer is more complex than that of normal cytokines. To Elucidate the mechanism of EV-mediated miRNA silencing in recipient cells is crucial for combating gliomas. Intercellular communication within the glioma microenvironment is, at least partially, mediated by EVs containing miRNA. EV-secreted miRNAs are thought to be endocytosed, after which they may exert oncogenic effects in glioma (Rooj et al., 2016). Thus, EV-secreted miRNAs may provide novel diagnostic and prognostic biomarkers, and may even serve as therapy targets of glioma.

In one study, primary human glioma cell-released EVs were isolated and used to treat primary mouse microglia. Mouse glioma cells were implanted into mice brains, after which EV release and uptake by microglia/macrophages were investigated. Proliferation of microglia/macrophages and production of immune suppressing cytokines were observed following uptake of glioma-EVs. Specifically, miR-451 and miR-21 in glioma-EVs were found to be efficiently transferred to microglia, while c-Myc mRNA was repressed in microglia at the same time. c-Myc has been shown to be a target of miR-451/miR-21, suggesting that EV-mediated miRNA silencing may be a functional link between glioma and microglia. Interestingly, in the brain, the release of EVs from glioma cells and the subsequent uptake by microglia/macrophages were directly visualized by researchers, as microglia/macrophages showed increased miR-21 and reduced c-Myc mRNA in the tumor-bearing brain (van der Vos et al., 2016). The interesting point is that the specific role of miR-21 (Moore and Zhang, 2010) and miR-451 in glioma is totally opposite. Circulating miR-21 dramatically increased in serum from glioma patients (Ivo D’Urso et al., 2015) and positively co-localized with angiogenesis marker VEGF in glioma tissue (Hermansen et al., 2016). In glioma cells, miR-21 exerted its oncogenic effects by promoting invasion (Gabriely et al., 2008) and repressing apoptosis (Quintavalle et al., 2013). Therefore, several groups already designed some tactics to fight against glioma by inhibiting miR-21 (Belter et al., 2016; Seo et al., 2019). However, miR-451 also functions as a tumor suppressor of glioma by repressing some key malignant cellular functions of glioma, such as proliferation, migration and apoptosis (Godlewski et al., 2010; Nan et al., 2010; Guo H. et al., 2016). Thus, EVs could be “oncogenic factor” or “tumor suppressor” by secreting different microRNAs (miR-21 or miR-451). It is critical to identify the specific inducer of secretion of oncogenic or anti-tumor miRNAs from EVs, which is a potential key step to elucidate the crosstalk between microglia and glioma cells in the tumor microenvironment.

miRNA could also directly mediate communication between glioma and microglia in the brain without contributions from EVs. Another study (Karthikeyan et al., 2018) demonstrated that the TGF-β signaling pathway in glioma-associated microglia participated in glioma pathogenesis. TGF-β activated in malignant glioma was shown to regulate glioma progression by modulating the Mothers against decapentaplegic homolog (SMAD) signaling pathway. SMAD4 has been shown to be a direct target of miR-146a, which was repressed in microglia treated with glioma-conditioned medium. Ectopic expression of miR-146a inhibited SMAD4 and another tumor-suppressor, MMP9, in microglia, and inhibited microglia migration towards glioma-conditioned medium. Moreover, glioma cell proliferation was reduced when treated with conditioned medium from SMAD4 downregulated or miR-146a up-regulated microglia. In addition, the tumor suppressive role of miR-146a also has been extensively studied in glioma cells (Mei et al., 2011; Lu et al., 2016; Li et al., 2018). A functional polymorphism in the pre-miR-146a gene [AG(C polymorphism (rs2910164)] is associated with risk and prognosis in glioma (Permuth-Wey et al., 2011). The therapeutical role of secreted miR-146a from marrow stromal cell (MSC) was also validated in glioma (Katakowski et al., 2013). Taken together, miR-146a-mediated crosstalk between microglia and glioma promotes microglial migration and glioma cell viability, which is a promising target with dual effects (microglia and glioma cells).

### Two Well-Studied miRNAs in Microglia

#### miR-124

miR-124 overexpression repressed motility and phagocytosis of apoptotic cells of microglia and caused residual apoptotic cell bodies to accumulate in the optic tectum. This *in vivo* study showed that miR-124 is essential for microglia development (Svahn et al., 2016). miR-124 is also required for maintaining the “resting” state of mouse monocytes. The *ex vivo* transfection of chitosan/miR-124 polyplex particles into rat microglia resulted in decreased inflammatory cytokine secretion, including ROS, TNF-α, and MHC-II. miR-124 particles were internalized by OX42- (rat macrophage marker) positive macrophages/microglia and reduced the number of ED-1- (rat macrophage marker) positive macrophages in the SCI (Louw et al., 2016).

Cocaine was found to inhibit miR-124 levels in microglia *in vitro*. miR-124 was also downregulated in isolated microglia from cocaine-administered mice (Guo M. L. et al., 2016). Cocaine-induced DNA methylation in the promoter region of miR-124 precursors, suggesting that cocaine exposure repressed miR-124 expression by inducing DNA methylation in the miR-124 promoter, which ultimately activates microglia in brain. Thus, epigenetic modification (e.g., DNA methylation in the promoter region) of miR-124 is a promising therapeutic approach in microglia-mediated cocaine addiction. D-4F (an apolipoprotein-A1 mimetic peptide) induced miR-124a and reduced matrix metalloproteinase-9, TNF-α, and TLR-4 in primary cortical neurons and microglia isolated from ischemic mice (Ning et al., 2017). The anti-inflammatory effects of D-4F were repressed in miR-124 knockdown primary neurons/microglia. D-4F treatment of type one diabetes mellitus (T1DM)-stroke increased miR-124 and promoted M2 macrophage polarization-mediated anti-inflammatory effects. miR-124 is highly expressed in neuronal cells but only shows a basal level in microglia. miR-124 could promote neuronal differentiation, modulate microglial activation, and keep microglia in a quiescent state. miR-124 also shifted pro-inflammatory M1 microglia/macrophages toward the anti-inflammatory M2 phenotype in the sub-acute phase of stroke (Hamzei Taj et al., 2016a), which suggests the ability to restore neurological deficits. Thus, miR-124 administration is a potential treatment for increasing rehabilitation change of stroke.

Morphine administration or inhibition of acetylcholine induced miR-124 expression in microglia and bone marrow-derived macrophages (Qiu et al., 2015). On the contrary, miR-124 promoted the inhibitory effects of morphine on innate immunity by suppressing TRAF6 and a subunit of NF-κB p65. Moreover, two additional transcriptional factors, Active protein (AP)-1 and cAMP response element-binding protein (CREB), were shown to repress miR-124, whereas p65 induced miR-124 transcription by targeting the miR-124 promoter. Therefore, a feedback loop seemingly formed between miR-124 and p65. Modulating miR-124 is a promising target for preventing opioid-induced damage to microglia. The expression of miR-124 increased in M2 microglia. Furthermore, overexpression of miR-124 repressed the production of proinflammatory cytokines. A co-culture system of microglia and neurons (Yu et al., 2017) showed that M2 microglia protected neurons from injury. miR-124 was also shown to bind to the 3′-UTR of CCAAT enhancer binding protein alpha (C/EBP-α). *In vivo*, miR-124 repressed expression of C/EBP-α and restored brain injury in ICH mice. Therefore, miR-124 prevents inflammatory damage in ICH by promoting M2 microglia polarization. In summary, miR-124 may represent a new therapeutic target for treatment of ICH.

**Figure 5:**
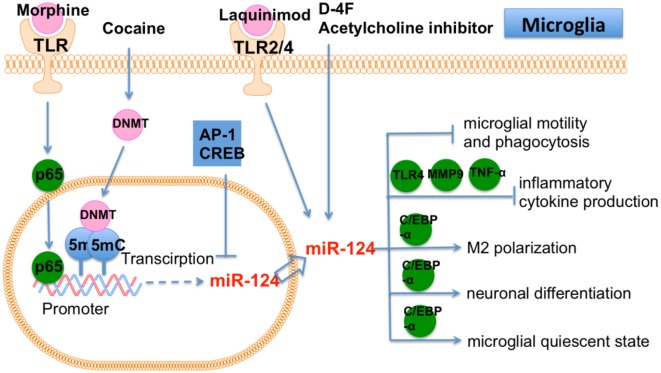
The role of miR-124 in microglia. Cocaine, D-4F, Morphine and Laquinimod induce miR-124 expression in microglia. miR-124 affects many cellular function of microglia, including motility, phagocytosis, polarization, differentiation and quiescent state.

Laquinimod is an oral treatment for MS. Laquinimod reduced brain atrophy and prevented the decline of activated microglia induced by miR-124a (Mishra et al., 2014). Laquinimod also blocked several proinflammatory pathways in microglia, including JNK and AKT pathways. It was further demonstrated that miR-124 inhibited PU.1, an essential downstream target of C/EBP-α, leading these microglia to a quiescent CD45 (low), MHCII (low) phenotype. miR-124 decreased in microglia in experimental autoimmune encephalomyelitis (EAE). Peripheral infusion of miR-124 in EAE led to systemic deactivation of macrophages and T cells, which eventually ameliorated EAE. In addition, miR-124 inhibition in microglia led to the activated state (Ponomarev et al., 2011). miR-124 is required for microglia status in CNS (Figure 5).

The expression level of miR-124 in microglia seemed to reflect the physiological state of brain and the progression of CNS diseases. It would be worthwhile to further explore these observations and elucidate its potential as biomarker for CNS diseases. Based on previous findings that have been summarized in the miR-124 related literatures, the specific function of miR-124 seems quite versatile in microglia depending upon different physiological and pathological conditions. This suggests that the role of miR-124 is contextual and mainly depends on the microenvironment of the brain in different brain tissues. Therefore, to further illuminate the regulatory role of miR-124 in brain, it will be necessary to pay more attention to the interaction between microglia and other cellular components in brain. In addition, miR-124 targets multiple targets simultaneously in brain (Figure 5). It is known that CNS diseases are often induced by multifactorial causes and the related treatment needs to act on a group of targets. Therefore, miR-124 is a potential therapy for the complexity of pathophysiological events that have been induced by multifactorial CNS diseases. Taken together, miR-124-based treatment is a potential therapy that may replace single-target therapy for the multifactorial CNS disease, which is definitely worth further investigation.

#### miR-155

LPS, a proinflammatory TLR4 ligand, greatly induced the expression of miR-155 in primary murine microglia (Woodbury et al., 2015). LPS-induced neurogenic defects and microglial activation were restored in miR-155 knockout mice. In contrast, microglial proliferation was induced in miR-155 knockin mice. miR-155 overexpression in microglia was also shown to cause neurogenic effects on neural stem cell (NSC) amoeboid morphology in the dentate gyrus (DG). Therefore, miR-155 mediated LPS-induced neuroinflammation by modulating microglia. Another study showed that ectopic expression of miR-155 caused endothelial hyperplasia and up-regulated microglial proliferation/activation, while miR-155 knockout mice displayed vascular defects. Furthermore, miR-155 was induced and microglia were activated in an oxygen-induced retinopathy (OIR) mouse model, which is characterized by abnormal angiogenesis. In addition, miR-155 knockout mice showed repressed microglial activation and abnormal vessel growth following ischemic insult. The CYR61 cysteine rich angiogenic inducer 61 (CCN1) gene is a direct target of miR-155. CCN1 encodes an extracellular matrix-associated integrin-binding protein that is essential for normal angiogenesis. Importantly, CCN1 knockout or double CCN1/miR-155 knockout mice showed retinal vascular dysfunction (Yan et al., 2015), highlighting an important role for miR-155-CCN1 in microglial activation and vascular abnormality.

Brains from 3xTg AD mice (a typical AD model) showed high levels of miR-155 expression (Guedes et al., 2014). Enhanced microglial activation was also observed in brains from AD mice. miR-155 and c-Jun were found to be increased in AD mice and Ab-activated microglia, which eventually resulted in the secretion of inflammatory cytokines. In addition, a c-Jun inhibitor was shown to repress miR-155 expression in activated microglia. Thus, miR-155 is a promising target to control neuroinflammation in AD.

LPS-repressed suppressor of cytokine signaling 1 (SOCS-1) is involved in the suppression of inflammation and is a direct target of miR-155 (Cardoso et al., 2012). Knockdown of miR-155 induced SOCS-1 expression and led to downregulation of iNOS and nitric oxide production. Conditioned medium from a miR-155 knockdown microglia culture was shown to reverse neuronal cell death. Thus, miR-155 has a pro-inflammatory effect in microglia *via* repression of SOCS-1. LPS treatment also leads to miR-155 up-regulation in BV2 cells. LPS repressed promoted apoptosis of BV2 cells and induced the production of pro-inflammatory cytokines. Meanwhile, miR-155 knockdown in BV2 cells rescued LPS-induced damage. Receptor of activated protein C kinase (RACK) 1 has been shown to be a target of miR-155 (Yin et al., 2017).

**Figure 6:**
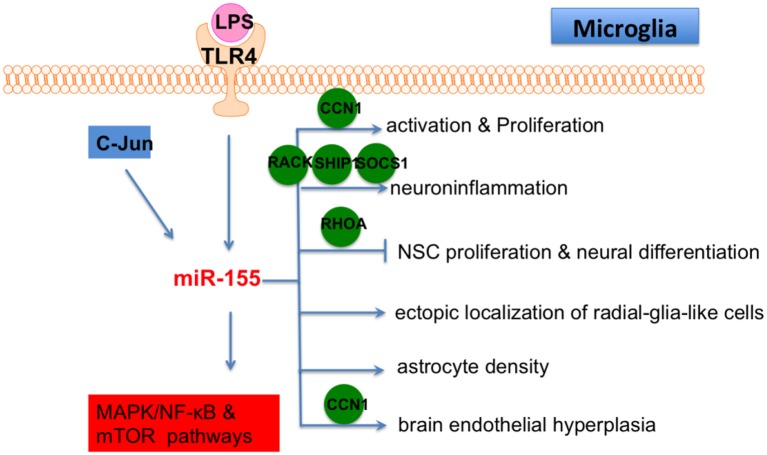
The role of miR-155 in microglia. Lipopolysaccharide (LPS)/TLR4 and C-Jun are two upstream regulators of miR-155 in microglia and MAPK/NF-κB/mTOR are downstream pathways of miR-155 in microglia. In addition, miR-155 also affects microglial activation, proliferation, inflammation, differentiation and fuction of other cells in brain (ectopic localization of radial-glia-like cells, astrocyte density and brain endothelial hyperplasia) by directly targeting various genes, including CCN1, RACK, SHIP, SOCS1, RHOA and CCN1.

Taken together, these studies showed that miR-155 is a central regulator in CNS-related inflammation through its ability to affect microglia. Therefore, miR-155 should be strongly considered as a therapeutic target of neuroinflammatory disease in the future (Figure 6) because it regulates multiple functions of microglia, including activation, proliferation, inflammation and interacts with other cells in brain, such as NSC, astrocyte and endothelial cells. Collectively, these articles demonstrate that miR-155 is a pro-inflammatory factor in microglia and is necessary for the progression of neural diseases through repression of multiple targets, suggesting that miR-155 blockage is a potential neuroprotective target. An inhibition of miR-155 ameliorates the activity of dysfunctional microglia, implying that miR-155 has therapeutic effect on related neural diseases. As miR-155 associated pathogenesis is a novel concept in CNS diseases, many additional and essential mechanisms by which miR-155 regulates CNS pathogenesis will be discovered in the near future. With the systematical understanding of the specific role of miR-155 in microglia, we believe that miR-155 based therapy of neural diseases will become a promising direction.

## Conclusion

The roles of microglia in neuropathogenesis remain to be fully understood. However, there are several advantages of microglia in the potential treatment of neurodegenerative diseases, such as EAE, CNS viral infection, and neuroinflammation. Available from clinical patients and *in vitro* cultures, microglia are an excellent tool for future gene therapy approaches for neuropathogenesis. In addition, distinguishing between resident microglia and monocyte-derived macrophages in pathological brain tissue is essential for the treatment of CNS diseases. Essential roles of miRNAs in microglial activation, inflammation, and differentiation/polarization have been widely demonstrated. Given the well-established roles of microRNAs in modulating gene expression, miRNA-based therapies could be considered as an attractive strategy to improve/recover microglial function and regulate genes/pathways in neuropathogenesis. Nevertheless, above-mentioned literatures mostly investigated the role of individual miRNAs, a dominant miRNA that plays the central role in microglia activation is yet to be identified. It is possible that key miRNA player in microglia activation differs in various pathological environment. Therefore, the central miRNA player in microglia activation may be emerged by simultaneous functional evaluation of all miRNAs that involved in the context of a specific neurological disease. In addition, since miRNAs can bind multiple targets or signaling pathways simultaneously, the ‘fine-tuning’ capacity of miRNAs in microglia is also suitable for therapeutic intervention and maintaining brain homeostasis. However, we cannot ignore the potential “off-target effects” of miRNAs, which is a main obstacle for clinical application. In summary, the use of miRNAs as disease biomarkers or therapeutic targets in CNS represents a powerful and promising approach in the future.

## Author Contributions

YG, JT and WW drafted the manuscript. WH, XW, PZ and HK revised the manuscript.

## Conflict of Interest Statement

The authors declare that the research was conducted in the absence of any commercial or financial relationships that could be construed as a potential conflict of interest.
